# The Potential of Dietary Antioxidants from a Series of Plant Extracts as Anticancer Agents against Melanoma, Glioblastoma, and Breast Cancer

**DOI:** 10.3390/antiox10071115

**Published:** 2021-07-12

**Authors:** Mindaugas Liaudanskas, Vaidotas Žvikas, Vilma Petrikaitė

**Affiliations:** 1 Faculty of Pharmacy, Lithuanian University of Health Sciences, 44307 Kaunas, Lithuania; Mindaugas.Liaudanskas@lsmuni.lt (M.L.); Vaidotas.zvikas@lsmuni.lt (V.Ž.); 2 Institute of Cardiology, Lithuanian University of Health Sciences, 44307 Kaunas, Lithuania; 3 Faculty of Medicine, Lithuanian University of Health Sciences, 44307 Kaunas, Lithuania

**Keywords:** antioxidant, anticancer, marigold, sage, bearberry, eucalyptus, yarrow, apples

## Abstract

In modern society, cancer is one of the most relevant medical problems. It is important to search for promising plant raw materials whose extracts have strong antioxidant and anticancer effects. The aim of this study was to determine the composition of phenolic compounds in plant extracts, to evaluate their antioxidant and anticancer activity, and to find the correlations between those activities. Extracts of calendula, sage, bearberry, eucalyptus, yarrow, and apple were selected for the study. The phenolic compounds of these extracts were determined by the UPLC-ESI-MS/MS method and the antioxidant activity was evaluated in vitro by four different UV-VIS spectrophotometric methods (ABTS, DPPH, CUPRAC, FRAP). The anticancer activity of extracts was tested against melanoma IGR39, glioblastoma U-87, and triple-negative breast cancer MDA-MB-231 cell lines in vitro by MTT assay. The highest content of identified and quantified phenolic compounds was found in sage leaf extract and the lowest in ethanol eucalyptus leaf extract. The highest antioxidant activity was determined by all applied methods for the acetone eucalyptus leaf extract. The majority of extracts were mostly active against the melanoma IGR39 cell line, and possessed the lowest activity against the glioblastoma U-87 cell line. Acetone extract of eucalyptus leaf samples exhibited the highest anticancer activity against all tested cell lines. Strong and reliable correlation has been found between antioxidant and anticancer activity in breast cancer and glioblastoma cell lines, especially when evaluating antioxidant activity by the FRAP method.

## 1. Introduction

Numerous scientific data confirm the benefits of natural antioxidants for human health [1]. A relationship between the consumption of plant-rich foods and the incidence of oncological [2], cardiovascular [3,4,5], and neurodegenerative diseases [6] has been established.

Evaluation of the antioxidant activity of plant extracts is extremely important. It provides a scientific basis for the use of herbal preparations for the prevention and treatment of oxidative stress disorders. Herbal extracts have a variety of compounds that act as antioxidants through different reaction mechanisms. Therefore, the scientific literature notes that performing a single test of antioxidant activity is not adequate, thus it is recommended to use at least two different methods [7]. Hence, we evaluated the antiradical and reductive activity of the plant raw material extracts by four different methods in vitro (ABTS, CUPRAC, DPPH, and FRAP). ABTS and DPPH assays are based on the ability of antioxidants to scavenge free radicals [8,9], whereas FRAP and CUPRAC assays measure the antioxidant reducing activity [10,11].

ABTS and CUPRAC methods allow to assess the antioxidant activity of both hydrophilic and lipophilic antioxidants, and their pH is close to that of body fluids [12,13]. An acidic medium (pH = 3.6) is used with the FRAP method [11], which can strongly influence the antioxidant properties of some natural antioxidants [14]. This method is not applicable to antioxidants whose mechanism of action is based on hydrogen transfer reactions [15]. Free DPPH radicals are soluble exclusively in organic solvents, which limits the evaluation of the antiradical activity of hydrophilic antioxidants by this method. One of the major limitations of the ABTS method is that any compound, even without being an antioxidant (e.g., various sugars and citric acid), that has a redox potential less than ABTS+ radical-cation may be involved in the reaction, and thus can distort the results.

Herbal extracts are multicomponent matrices that can contain thousands of different biologically active compounds that have a wide variety of biological effects on the human body. The search for promising plant-based raw materials and analysis of their chemical composition and biological activity is particularly important when developing new drugs for the prevention and treatment of various diseases. For this reason, it is important to assess de novo the chemical composition of herbal extracts used in traditional and folk medicine using modern research methods, in order to determine which raw materials have the highest antioxidant, anticancer, and other biological activity in vitro and in vivo. Extracts of plant raw materials for this study (eucalyptus, sage and bearberry leaves, yarrow grass, marigold flowers, and apples) were selected owing to their strong antioxidant and anticancer effects. González-Burgos et al. indicate that eucalyptus leaf extracts have strong antioxidant and neuroprotective effects [16]. Sage leaves are especially rich in phenolic compounds—natural antioxidants. Many studies have proven the anticancer effects of their extracts [17]. Scientific literature provides data on the anticancer effects [18,19,20,21] and antioxidant activity [22,23,24,25] of yarrow grass, calendula flowers, bearberry leaves, and apple fruit extracts.

One of the most important bioactive compounds accumulated in plants is phenolic compounds, which are classified as natural antioxidants. Oxidative stress causes changes in cellular metabolism associated with DNA, protein damage, and lipid peroxidation [26]. These changes can lead to inflammatory processes as well as cardiovascular, cancerous, and other diseases [27]. Phenolic compounds effectively neutralize reactive forms of oxygen and nitrogen [28], and are thus valuable in the prevention and treatment of many diseases. Studies on the qualitative and quantitative composition and biological activity of plant raw materials that accumulate phenolic compounds are important and relevant.

Cancer is one of the most relevant societal, scientific, and medical issues in modern society, as morbidity and mortality are constantly increasing throughout the world [29]. Although various cancer prevention measures and modern malignancy research methods are being implemented, it is still not possible to claim that cancerous diseases are defeated. Therefore, efforts have been made to find more effective treatments for cancer. Herbal extracts provide promising preparations for cancer prevention and treatment, the potential of which has not yet been fully evaluated and exploited. Researchers have shown that plant extracts, their isolated fractions, or individual components can inhibit cancer cell-stimulating enzymes, stimulate the production of anticancer enzymes, enhance the immune system response, and protect DNA and other cellular structural molecules through their antioxidant effects [30]. In this context, it is important to conduct anticancer activity studies of plant extracts by selecting extracts with the highest activity.

The relationship between the antioxidant effect of plant extracts and their anticancer activity is controversial [31,32]. There is information in the scientific literature regarding the association of antioxidant activity of plant extracts with their anticancer activity [33], but data denying this direct relationship have also been published [34]. For this reason, it is extremely important to evaluate the relationship between the chemical composition of specific plant extracts under investigation and their antioxidant and anti-cancer activity. To accomplish this task, a correlation analysis was performed, the results of which allow statistically reliable estimation of the existence of the above-mentioned relationships.

The main aim of this study is to determine the composition of phenolic compounds in selected plant extracts in order to evaluate their antioxidant and anticancer activity and correlation between the estimates of these parameters. The obtained research results will provide new knowledge about the qualitative and quantitative composition as well as antioxidant and anticancer activity of selected plant extracts widely used in traditional and folk medicine and their correlation. This up-to-date information will be valuable from a theoretical and practical point of view to identify plant extracts with the strongest antioxidant and anticancer activity that could be promising for further in vivo studies, the manufacture of pharmaceuticals for the prophylaxis and treatment of cancer, and their use in clinical practice.

## 2. Materials and Methods

### 2.1. Plant Material

*Eucalyptus globulus* Labill. and *Salvia officinalis* L. leaves, *Achillea* *millefolium* L. herb, and *Calendula* *officinalis* L. flowers (manufacturer JSC Acorus Calamus, Švenčionys, Lithuania) were bought in the local pharmacy. Dried material was ground to a powder using a mill (IKA^®^ A11 basic, Staufen im Breisgau, Germany). The apples samples (cultivar ‘Ligol’) were supplied by the Institute of Horticulture, Lithuanian Research Centre for Agriculture and Forestry, Babtai, Lithuania (55°60′ N, 23°48′ E). Each apple was cut into slices of equal size (up to 1 cm in thickness), and the stalks and seeds were removed. The slices were immediately frozen in a freezer (at −35 °C) with air circulation and then lyophilized with a Zirbus sublimator (ZIRBUS technology, Bad Grund, Germany) at the pressure of 0.01 mbar (condenser temperature, −85 °C). The lyophilized slices were ground to fine powder using a Retsch 200 mill (Haan, Germany). Loss on drying before analysis was determined by drying about 1 g of milled leaves in a moisture analyzer (Precisa HA 300, “Precisa Instruments AG, Dietikon, Switzerland) to complete evaporation of water and volatile compounds (drying temperature: 105 °C).

### 2.2. Cell Culture

Human melanoma cell line IGR39, human breast adenocarcinoma cell line MDA-MB-231, and human glioblastoma cell line U-87 (a kind gift from Dr. Manel Esteller, Bellvitge Biomedical Research Institute (IDIBELL)) were grown in DMEM Glutamax medium supplemented with 10% FBS and 1% antibiotics at 37 °C in a humidified atmosphere containing 5% CO_2_. All cell cultures routinely were grown to 70% confluence and trypsinized with 0.125% TrypLE™ Express solution before passage. They were used until passage 20.

### 2.3. Chemicals and Materials

All chemical solvents, reagents, and standards used were of analytical grade. Acetonitrile, acetone, and formic acid were obtained from Sigma-Aldrich GmbH (Buchs, Switzerland) and ethanol from JSC Vilniaus degtinė (Vilnius, Lithuania). 1,1-Diphenyl-2-picrylhydrazyl (DPPH^•^) radical, 2,2′-azino-bis(3-ethylbenzothiazoline-6-sulphonic acid) (ABTS), potassium persulfate, ammonium acetate, sodium acetate trihydrate, iron (III) chloride hexahydrate, and 2,4,6-tripyridyl-s-triazine (TPTZ) were obtained from Sigma-Aldrich (Steinheim, Germany). Neocuproine was purchased from Alfa Aesar (Kandel, Germany) and copper (II) chloride—from Carl Roth Carl Roth GmbH + Co. KG (Karlsruhe, Germany). Folin–Ciocalteu reagent, gallic acid monohydrate, sodium carbonate, aluminum chloride hexahydrate, and hexamethylenetetramine were purchased from Sigma-Aldrich GmbH (Buchs, Switzerland). All analytical standards for UPLC-ESI-MS/MS analysis were HPLC grade and were also purchased from Sigma–Aldrich (St. Louis, MO, USA), except for and isorhamnetin 3-O-glucoside, which was from Extrasynthese (Genay, France). Deionized water, produced by the Milli–Q^®^ (Millipore, Bedford, MA, USA) high-performance liquid chromatography water purification system, was used.

DMSO (≥99%, Ph. Eur. grade) was obtained from Sigma-Aldrich (St. Louis, MO, USA). 3-(4,5-dimethylthiazol-2-yl)-2,5-diphenyltetrazolium bromide (MTT, ≥97%) was purchased from Sigma-Aldrich (St. Louis, MO, USA).

All cell culture plastic ware was purchased from Thermo Fisher Scientific, Corning and Techno Plastic Products. TrypLE^TM^ Express, Dulbecco’s modified Eagle high glucose medium (DMEM Glutamax), fetal bovine serum (FBS), penicillin/streptomycin solution (100×), and phosphate buffered saline (PBS) were obtained from Gibco.

### 2.4. Extraction

The powder from *E. globulus* and *S. officinalis* leaves, *A. millefolium* herb, and *C. officinalis* flowers was extracted as described by González-Burgos et al. [16]. Raw material was soaked for 3 h in 40% or 70% (*v*/*v*) ethanol or acetone. Here, 40 and 70% ethanol was selected as an extractant based on literature data, providing the best extraction yield of phenolic compounds [35,36]. Further, 70% acetone was used for comparative studies of the extraction efficiency of eucalyptus leaf samples, as this solvent is proposed by some scientists as an effective extractant for the extraction of phenolic compounds [37,38].

Soaked raw material was transferred to a percolator, covered with extractant, and left to macerate (48 h). Then, it was percolated (rate 0.3 mL min^−1^) and high concentration extract (85% of total extract amount) was obtained. Low concentration extract was decanted and it was evaporated using a rotary evaporator (IKA^®^ HB 10, Staufen im Breisgau, Germany) up to 15% of the total liquid extract amount. The remaining part of the low concentration extract was transferred to a single container with high concentration extract. The organic phase of liquid extract was evaporated using a rotary evaporator and the remaining aqueous phase was lyophilised using lyophilisator Zirbus (Zirbus technology GmbH, Bad Grund, Germany) at 0.01 mbar pressure and condenser temperature of −85 °C.

The powder from lyophilized apples was extracted as described by Liaudanskas et al. 2014 [39]. For the extraction of apple samples, 70% ethanol was selected based on the results of apple flavonoid extraction efficiency studies published in this article. An amount of 2.5 g of lyophilized apple powder (exact weight) was weighed, added to 30 mL of ethanol (70%, *v*/*v*), and extracted in a Sonorex Digital 10 P ultrasonic bath (Bandelin Electronic GmbH & Co. KG, Berlin, Germany) for 20 min at 40 °C. The extract obtained was filtered through a paper filter; the apple lyophilizate on the filter was washed twice with 10 mL of ethanol (70%, *v*/*v*) in a 50 mL flask.

Extraction conditions (the solvent and the ratio are shown in Table 1.

### 2.5. UPLC–ESI–MS/MS Conditions

Qualitative and quantitative analysis of phenolic compounds was performed according to the previously validated and described UPLC–ESI–MS/MS method [16]. Separation of phenolic compounds was performed with Acquity H-class UPLC system (Waters, Milford, MA, USA) equipped with a triple quadrupole tandem mass spectrometer (Xevo, Waters, Milford, MA, USA) with an electrospray ionization source (ESI) to obtain MS/MS data. YMC Triart C18 (100 × 2.0 mm; 1.9 μm) column (YMC Europe GmbH, Dislanken, Germany) was used for analysis. The column temperature was maintained at 40 °C. Gradient elution was performed with mobile phase consisting of 0.1% formic acid water solution (solvent A) and acetonitrile (solvent B) with a flow rate set to 0.5 mL min^−1^. Linear gradient profile was applied as follows for solvent A: initially 95% for 1 min; to 70% over 4 min; 50% over 7 min; and 95% over 2 min. Negative electrospray ionization was applied for analysis: capillary voltage −2 kV, source temperature 150 °C, desolvation temperature 400 °C, desolvation gas flow 700 L h^−1^, and cone gas flow 20 L h^−1^. Collision energy and cone voltage were optimized for each compound separately.

### 2.6. Determination of Antioxidant Activity

ABTS^•+^ radical cation decolorization assay. An ABTS^•+^ radical cation decolorization assay was applied according to the methodology described by Re et al. (9)]. A volume of 3 mL of ABTS^•+^ solution (absorbance 0.800 ± 0.02) was mixed with 10 μL of the ethanol extract of apple leaves. A decrease in absorbance was measured at a wavelength of 734 nm after keeping the samples for 30 min in the dark. The regression equation for this assay was established to be *y* = 0.000084*x* − 0.002068; R^2^ = 0.998777.

DPPH^•^ free radical scavenging assay. The DPPH^•^ free radical scavenging activity was determined using the method proposed by Brand-Williams et al. [8]. DPPH^•^ solution in 96.3% *v*/*v* ethanol (3 mL, 6 × 10^−5^ M) was mixed with 10 μL of the ethanol extract of apple leaves. A decrease in absorbance was determined at a wavelength of 517 nm after keeping the samples for 30 min in the dark. The regression equation for this assay was established to be *y* = 0.000111*x* − 0.000709; R^2^ = 0.998024.

CUPRAC assay. The CUPRAC solution included copper (II) chloride (0.01 M in water), ammonium acetate buffer solution (0.001 M, pH = 7), and neocuproine (0.0075 M in ethanol) (ratio 1:1:1). Here, 3 mL of CUPRAC reagent was mixed with 10 μL of extracts. An increase in absorbance was recorded at λ = 450 nm. The regression equation for this assay was established to be *y* = 0.000048*x* − 0.001273; R^2^ = 0.998580 [10].

FRAP assay. The ferric reducing antioxidant power (FRAP) assay was carried out as described by Benzie and Strain (11).The working FRAP solution included TPTZ (0.01 M dissolved in 0.04 M HCl), FeCl_3_ × 6H_2_O (0.02 M in water), and acetate buffer (0.3 M, pH 3.6) at the ratio of 1:1:10. A volume of 3 mL of a freshly prepared FRAP reagent was mixed with 10 μL of the apple leaf extract. An increase in absorbance was recorded after 30 min at a wavelength of 593 nm. The regression equation for this assay was established to be *y* = 0.000128*x* − 0.034745; R^2^ = 0.998682.

Calculation of antioxidant activity of the extract. The antioxidant activity of extracts was calculated from the Trolox calibration curve and expressed as μmol Trolox equivalent (*TE*) per gram of absolutely dry weight (DW). *TE* was calculated according to the following formula:

(1) $$TE=\frac{c\times V}{m},$$

*c*—the concentration of Trolox established from the calibration curve (in μM); *V*—the volume of leaf extract (in L); *m*—the weight (precise) of lyophilized leaf powder (in g).

### 2.7. Cell Viability Assay

Cell viability was studied using the method of MTT. Here, 100 μL of cells was seeded in 96-well plates in triplicate (5000 cells/well) and incubated at 37 °C for 24 h. Then, serial double dilutions of tested extracts (from 10 mg/mL to 0.156 mg/mL) were made in microplates. Cells treated only with medium containing 1.0% of ethanol served as a negative control. Free medium without cells was used as a positive control. After 72 h incubating at 37 °C, the cell growth medium in all the wells was replaced with the new one containing 0.5 mg/mL of MTT. After 4 h, the liquid was aspirated from the wells and discarded. Formazan crystals were dissolved in 100 μL of DMSO, and absorbance was measured at a test wavelength of 490 nm and a reference wavelength of 630 nm using a multi-detection microplate reader. The experiments were repeated three times independently and the results were given as means ± SD.

Applying Hill fit to compound dose–cell metabolic activity (absorbance) curves, the effective concentration (*EC*_50_) values, reducing cell viability by 50%, were calculated.

### 2.8. Statistical Analysis

All the data obtained from this study were analysed statistically using SPSS™ software for Windows, Version 20.0 (SPSS Inc., Chicago, IL, USA) and the Microsoft Excel software package (Microsoft Corp, Redmond, WA, USA). Data are presented as mean ± standard error (S.E.) of at least three independent experiments. The correlation between antioxidant and anticancer activity was assessed by calculating Pearson‘s coefficient *r* and its statistical reliability. Correlation was considered as very strong when *r* = 0.90–0.99 (positive) or −0.99–(−0.90) (negative); strong when *r* = 0.70–0.89 (positive) or −0.89–(−0.70) (negative); and moderate when *r* = 0.40–0.69 (positive) or −0.69–(−0.40) (negative).

## 3. Results and Discussion

### 3.1. Extract Composition

Qualitative and quantitative phenolic composition of tested extracts was investigated using the UPLC–ESI–MS/MS method (Table 2).

The total amount of identified and quantified phenolic compounds in the tested extracts ranged from 151.79 ± 6.21 mg/g (ethanol extract of eucalyptus leaves) up to 1931.95 ± 80.37 mg/g (sage leaf extract). The total amount of phenolic compounds in eucalyptus leaf samples extracted with different extraction solvents (acetone and ethanol) did not differ statistically significantly and was significantly lower than that of other plant raw material extracts (Table 2); the extraction solvent had no statistically significant effect on the extraction yield.

The UPLC–ESI–MS/MS analysis of selected plant extracts showed that flavonoid compounds were the major component among all identified phenolic compounds, and 17 different flavonoids were identified and quantified. The total amount of flavonoids detected in the extracts of the analyzed samples varied significantly. The highest total amount of the identified and quantified compounds of the flavonoid group was found in marigold blossom extracts (1043.78 ± 47.5 mg/g), and the lowest in sage leaf extracts (33.52 ± 1.42 mg/g).

The most abundant group of flavonoids in the investigated extracts is quercetin derivatives. The scientific literature indicates that quercetin and its glycosides have a broad biological activity. They might reduce the risk of cardiovascular diseases [40], metabolic disorders [3], and certain types of cancer [41]. Aglycone quercetin and its glycosides—avicularin, hyperoside, and rutin—were identified after analysis of selected plant extracts. Aglicones and glycosides of kaempherol and isorhamnetine luteolin glycosides, apigenin and its glycoside vitexin, and other flavonoids were detected in the extracts of the studied plant raw materials. The amount of flavonoids identified and quantified in the extracts of different plant raw materials varied widely. The acetone extract of eucalyptus leaf samples was dominated by phloridzin and the ethanol extract by quercetin. Marigold leaf blossom extracts were dominated by isorhamnetin 3-O-rutinoside, whereas all of the eucalyptus leaf sample extracts and yarrow grass extract extracts did not even contain this compound. Apigenin predominated in the extracts of sage leaves and yarrow grass. Quercetin group glycosides avicularin and hyperoside dominated in the extracts of bearberry berries and apples, and apigenin was not identified in these extracts. The obtained results confirm the claims of other scientists that the plant species has a great influence on the diversity of the composition of biologically active compounds [42].

Another group of phenolic compounds found in tested plant extracts was phenolic acids. The vast majority of the identified phenolic acids were hydroxycinnamic acid derivatives. Only one derivative of hydroxybenzoic acid, syringic acid, was identified. The highest total amount of identified and quantified phenolic acids (1898.43 ± 85.21 mg/g) was found in sage leaf extracts, and the lowest (81.93 ± 3.12 mg/g) in ethanol eucalyptus leaf extracts. Chlorogenic acid was predominant in most of the plant material sample extracts studied for a wide range of potential health benefits, including its strong antioxidant [43], anti-diabetic [44], anti-carcinogenic [45], anti-inflammatory [46], and anti-obesity impacts [47]. An exception was the sage leaf extracts, which were large enriched with rosmarinic acid and contained a relatively low amount of chlorogenic acid (Table 2).

Quinic acid (cyclitol), which is not classified as phenolic compound, was identified and quantified in the tested extracts. This acid was predominantly found in most of the tested plant raw materials, except for marigold and sage leaf extracts (Table 2).

### 3.2. Antioxidant Activity

**Figure 1:**
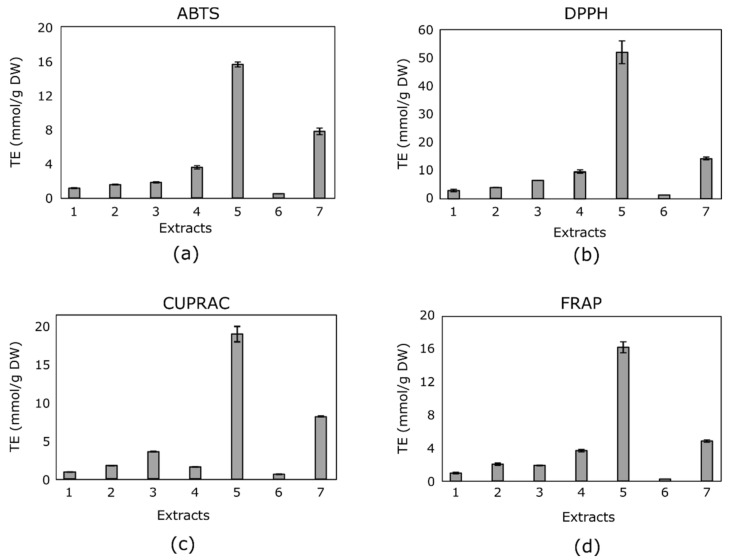
The antioxidant activity of all tested extracts by the (**a**) ABTS, (**b**) DPPH, (**c**) CUPRAC, and (**d**) FRAP methods.

Acetone extract of eucalyptus leaf samples exhibited the strongest antiradical activity assessed by the ABTS and DPPH methods (1.56 ± 0.03 mmol TE/g DW and 5.20 ± 0.40 mmol TE/g DW, respectively) and reducing activity assessed by the CUPRAC and FRAP methods (1.98 ± 0.11 mmol TE/g DW and 16.26 ± 0.67 mmol TE/g DW, respectively). The lowest antiradical and reducing activity in vitro, as assessed by all methods applied, was determined by examination of apple sample extracts; Figure 1.

The differences in the results obtained may have been influenced by a variety of factors—different media or pH [48], different lipophilic-hydrophilic properties of different antioxidants [49], and differences in qualitative and quantitative composition between the extracts and mechanisms of applied methods.

The antioxidant activity of plant extracts is determined by a complex of biologically active compounds. The scientific literature indicates that one of the strongest antioxidants that can determine the anti-radical and reducing properties of extracts is phenolic compounds [50]. However, in our study, there was no clear relationship between the quantitative composition of phenolic compounds and the in vitro antioxidant activity of the plant extracts tested. Contrary to expectations, extracts of sage leaves and calendula flowers, which contained the highest levels of identified and quantified compounds, did not exhibit strong antioxidant activity. In contrast, eucalyptus leaf extracts, which contained small amounts of phenolic compounds, exhibited particularly strong antiradical and reductive activity in vitro. These results can be explained by the fact that only part of the phenolic compounds responsible for such potent antioxidant activity of eucalyptus leaf extracts have been identified in vitro. The antioxidant activity of these extracts may have been due to phenolic compounds other than the antioxidants that we did not detect.

In order to evaluate the correlation strength between the in vitro antioxidant activities of tested plant extracts by different methods, the Pearson correlation coefficient was calculated (Table 3). A strong correlation between the antiradical and reducing activity was established. A strong correlation was found between the in vitro antioxidant activity assessed by ABTS and DPPH. Such results can be explained by the fact that the mechanism of action of both methods is based on the binding (inactivation) of simulated synthetic free radicals—ABTS^•+^ radical cation or DPPH^•^ free radical [8,9]. The weakest correlation was found between antioxidant activity assessed by FRAP and CUPRAC methods. Such data are quite unexpected, as both methods are based on the same mechanism of action—they determine the reducing activity of extracts or other solutions. Such a result could be explained by the differences between these methods. The pH of the reaction medium is neutral (pH = 7) with the CUPRAC method and acidic (pH = 3.6) with the FRAP method [10,11]. The pH of the medium is a critical factor in assessing the in vitro antioxidant activity of plant antioxidants, especially phenolic compounds [36]. Various scientific studies have shown that the antioxidant activity of phenolic compounds is highly dependent on the pH of the medium [14]. The antioxidant activity of phenolic compounds is mediated by functional hydroxyl groups [39]. As the pH of the medium decreases, the dissociation process of the hydroxyl group weakens, leading to a lower antioxidant activity of the phenolic compounds [10]. The antioxidant activity of plant antioxidants based on hydrogen atom transfer reactions (thiols, carotenoids) cannot be determined by the FRAP method [40,41]. Perhaps, the above mentioned differences between the FRAP and CUPRAC methods may have led to a slightly weaker correlation between their in vitro antioxidant activity.

### 3.3. Anticancer Effect

**Figure 2:**
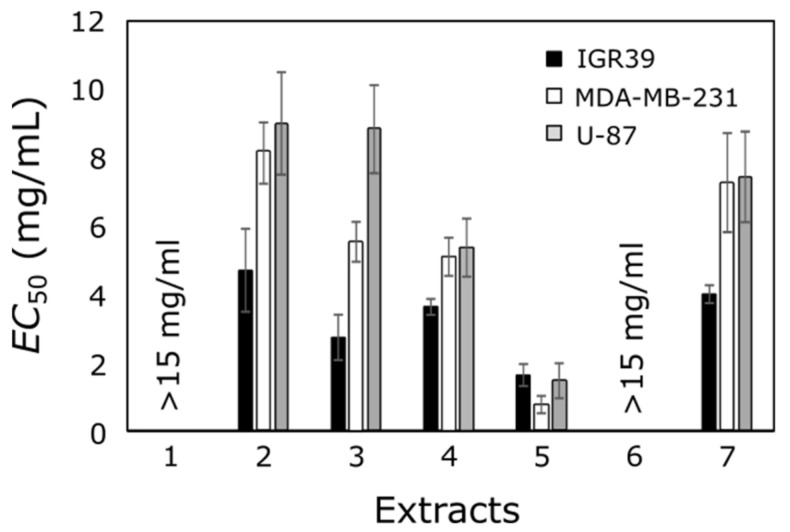
The *EC*_50_ values of tested extracts against IGR39, MDA-MB-231, and U-87 cancer cell lines.

The effect of the tested extracts on cancer cell viability was variable (Figure 2). Six out of eight extracts possessed a cytotoxic effect against all cancer cell lines. Extract of apples and extract of calendula flowers did not possess anticancer activity at the tested concentration (up to 15 mg/mL). Sak et al. in their experiments in vitro against human melanoma SK-MEL-2 cells also observed relatively low activity of marigold extract [51]. A weak antiproliferative effect on several breast cancer cell lines of extracts made from apple peels was established by other scientists too [52].

The majority of extracts were mostly active against the melanoma IGR39 cell line, and possessed the lowest activity against the glioblastoma U-87 cell line. However, this cell line is constitutively resistant to many chemotherapeutic agents owing to over-expression of P-glycoprotein and its stemness properties [53]. Only one extract showed a cell viability reducing effect against glioblastoma cells at lower than 2 mg/mL concentration. Triple-negative breast cancer cell line MDA-MB-231 was also quite resistant to the tested extracts. Only one extract showed an anticancer effect on these cells at lower than 2 mg/mL concentration (dried eucalyptus leaf acetone extract). It could be explained that this cell line does not have estrogen, progesterone, and HER-2 receptors, and is characterized by a more aggressive nature than other types of breast cancer cells [54].

Acetone extract of eucalyptus leaf samples exhibited the highest anticancer activity against melanoma, triple-negative breast cancer, and glioblastoma cell lines (1.6 ± 0.3 mg/mL, 0.8 ± 0.3 mg/mL, and 1.5 ± 0.5 mg/mL, respectively). These results are consistent with the antioxidant activity, as this extract showed the highest antioxidant effect by all the methods used (Figure 1). The lowest anticancer effect was determined for the same extracts that showed the lowest antioxidant activity (extract of apples and extract of calendula flowers).

**Figure 3:**
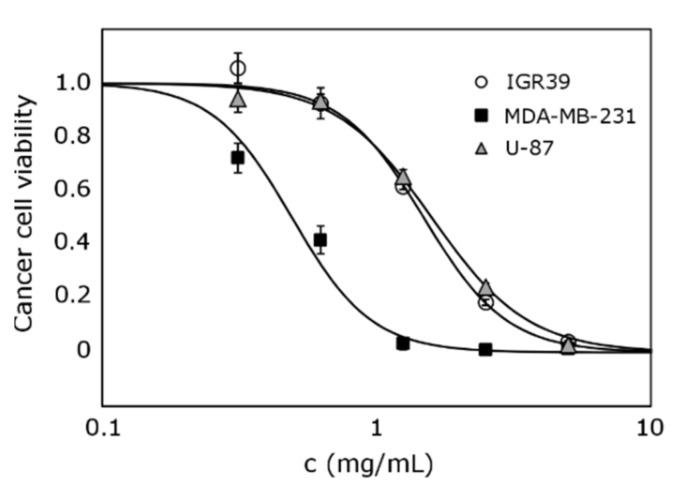
Comparison of extract 6 activity against IGR39, MDA-MB-231, and U-87 cell lines.

The most active extract, made from eucalyptus leaves with acetone, possessed different activity on tested cell lines (Figure 3). The highest anticancer effect was observed on the triple-negative breast cancer cell line MDA-MB-231.

However, contrary to expectations from antioxidant activity assays, ethanol extract of eucalyptus leaves did not exhibit high anticancer activity. In contrast to other eucalyptus leaf extracts made with acetone, this extract was from 2.4 to 9.5 times less active against different cell lines. These results can be explained in that the anticancer effect is not necessarily related to antioxidant properties of active substances, and such a phenomenon was observed previously with different substances with antioxidant activity in vitro [55]. In our study, there was no clear relationship between the quantitative composition of phenolic compounds and the in vitro anticancer activity of the tested plant extracts.

Apigenin, caffeic acid, catechin, chlorogenic acid, quercetin, kaempherol, and other compounds that are widely known to possess activity against cancer cells [56,57,58,59] were not found in the most active extract or were detected only at quite low amounts (Table 2). This could mean that this extract contains other substances that could be responsible for its anticancer effect, or the complex of all substances could possess strong synergistic activity. In contrary, some moderately active extracts contained the above-mentioned compounds in high levels, but still did not show high activity against tested cancer cell lines, and their effect was different between them. It was noticed previously by other scientists, that some compounds, such as apigenin, may show different activity depending on cell prototype [60].

Our results showed that anticancer activity could be related to antioxidant activity of extracts (Table 4).

A strong and reliable correlation was found between antioxidant and anticancer activity in breast cancer and glioblastoma cell lines, especially when evaluating antioxidant activity by the FRAP method. The same correlation between antioxidant and anticancer activity in melanoma cells was established as moderate; however, it was not found to be reliable. This could mean that the mechanism of anticancer activity in melanoma cells could be different and is less related to the scavenging free radicals or the antioxidant reducing activities.

## 4. Conclusions

The extracts of calendula, sage, bearberry, eucalyptus, yarrow, and apple widely used in traditional and fold medicine contain 23 individual phenolic compounds, including 17 flavonoids. The highest amount of identified and quantified compounds was found in sage leaf extract. Rosmarinic acid was predominant in these extracts, while chlorogenic acid was predominant in the extracts of yarrow grass and apple tree fruits. Quinic acid, a non-phenolic compound, was identified and quantified and was found in the highest amount in the extracts of bearberry leaves.

The highest antioxidant activity in vitro was determined using all applied methods in acetone extracts of eucalyptus leaves. The weakest antiradical and reductive activity in vitro by all methods was detected in apple extracts.

Six out of eight extracts possessed cytotoxic effect against triple-negative breast cancer (MDA-MB-231), melanoma (IGR39), and glioblastoma (U-87) cell lines. Acetone extract of eucalyptus leaf samples exhibited the highest anticancer activity against all tested cell lines. A strong and reliable correlation was found between antioxidant and anticancer activity in breast cancer and glioblastoma cell lines, especially when evaluating antioxidant activity by the FRAP method.

## Figures and Tables

**Table 1:** Extracts prepared for chemical composition and biological activity analysis.

No.	Raw Material	Extraction Solvent	Concentration of Extraction Solvent, % (*v*/*v*)	The Ratio of Extraction Solvent to Raw Material
1	Dried calendula flowers	Ethanol	40	1:5
2	Dried sage leaves	Ethanol	40	1:5
3	Dried yarrow herb	Ethanol	40	1:5
4	Dried bearberry leaves	Ethanol	70	1:20
5	Dried eucalyptus leaves	Acetone	70	1:2
6	Lyophilized apples	Ethanol	70	1:20
7	Dried eucalyptus leaves	Ethanol	40	1:2

All extracts were filtered through a membrane filter with a pore size of 0.22 μm (Carl Roth GmbH, Karlsruhe, Germany).

**Table 2:** Chemical composition of extracts. Grey colour—tested substance was not detected, blue colour—very high amount of tested substance was detected.

Active Substances	Extracts						
**mg/g DW**	**1**	**2**	**3**	**4**	**5**	**6**	**7**
Apigenin	12.99 ± 0.47	20.32 ± 0.77	92.88 ± 4.32				
Aviculiarin				276.9 ± 10.23	0.75 ± 0.03	118.61 ± 5.42	2.58 ± 0.09
Caffeic acid	50.65 ± 2.03	30.74 ± 1.22	112.38 ± 4.93				0.06 ± 0.01
(+)-Catechin			0.03 ± 0.01	220.2 ± 10.41	0.32 ± 0.01	70.09 ± 2.98	0.03 ± 0.01
Chlorogenic acid	206.16 ± 8.35	11.84 ± 0.48	538.5 ± 24.11	184.05 ± 7.36	94.97 ± 4.01	677.6 ± 30.23	80.24 ± 2.47
p-Coumaric acid		16.24 ± 0.05					1.56 ± 0.05
Galangin							0.90 ± 0.04
Hyperoside	93.96 ± 4.50		7.35 ± 0.31	273.6 ± 13.26	2.65 ± 0.07	114.24 ± 5.63	1.59 ± 0.06
Isorhamnetin	241.65 ± 11.0	0.40 ± 0.02	10.60 ± 0.44	1.81 ± 0.07			0.66 ± 0.02
Isorhamnetin 3-*O*-glucoside	171.15 ± 8.03		0.78 ± 0.03	17.92 ± 0.82			
Isorhamnetin 3-*O*-rutinoside	320.41 ± 15.3	2.26 ± 0.09		82.54 ± 4.03		1.66 ± 0.06	
Kaempherol	1.35 ± 0.06	0.15 ± 0.01	0.47 ± 0.02	0.49 ± 0.02	0.22 ± 0.01		1.49 ± 0.05
Kaempherol 3-*O*-glucoside	8.79 ± 0.37	1.17 ± 0.05	0.12 ± 0.01	11.17 ± 0.47	0.70 ± 0.02	ND	0.32 ± 0.01
Luteolin 7-*O*-glucoside	7.54 ± 0.27			9.74 ± 0.41			
Orientin	0.48 ± 0.02	0.82 ± 0.03	14.60 ± 0.67	2.99 ± 0.14			3.75 ± 0.17
Phloridzin			3.83 ± 0.14	3.30 ± 0.12	35.51 ± 1.42	64.90 ± 3.01	20.93 ± 0.88
Quercetin	32.14 ± 1.47		5.76 ± 0.20	38.20 ± 1.70	2.59 ± 0.10	1.61 ± 0.06	28.40 ± 1.33
Quinic acid	381.56 ± 16.3	4.09 ± 0.18	2342.1 ± 101.4	7824.8 ± 350.6	703.6 ± 32.03	5398.6 ± 213.36	627.94 ± 28.9
Rosmarinic acid	0.39 ± 0.01	1799.2 ± 80.5	7.17 ± 0.29	9.80 ± 0.42	0.34 ± 0.02	106.35 ± 4.69	0.07 ± 0.01
Rutin	153.32 ± 7.12	6.87 ± 0.29	29.21 ± 1.20	71.97 ± 2.98	12.53 ± 0.46	22.92 ± 1.03	7.30 ± 0.28
Syringic acid	48.59 ± 1.97	40.45 ± 1.76					
Tiliroside		1.21 ± 0.04		3.01 ± 0.11	ND	0.65 ± 0.02	0.15 ± 0.01
Vitexin		0.32 ± 0.02	1.46 ± 0.05		1.90 ± 0.08	ND	1.76 ± 0.06

**Table 3:** Correlation between the different methods applied for testing antioxidant activity in this research.

		ABTS	DPPH	CUPRAC	FRAP
ABTS	Correlation coefficient	1	0.972 **	0.981 **	0.976 **
	*p* value (two-tailed)		0.000	0.000	0.000
DPPH	Correlation coefficient	0.972 **	1	0.976 **	0.997 **
	*p* value (two-tailed)	0.000		0.000	0.000
CUPRAC	Correlation coefficient	0.981 **	0.976 **	1	0.971 **
	*p* value (two-tailed)	0.000	0.000		0.005
FRAP	Correlation coefficient	0.976 **	0.997 **	0.971 **	1
	*p* value (two-tailed)	0.000	0.000	0.005	

** Correlation is significant at the 0.01 level (two-tailed).

**Table 4:** Correlation between antioxidant and anticancer activity of tested plant extracts.

		Antioxidant Activity, mmol TE/g DW			
**Anticancer Activity**		**ABTS**	**DPPH**	**CUPRAC**	**FRAP**
IGR39	Correlation coefficient	−0.583	−0.558	−0.517	−0.600
	*p* value (two-tailed)	0.129	0.151	0.190	0.116
MDA-MB-231	Correlation coefficient	−0.791 *	−0.793 *	−0.744 *	−0.803 **
	*p* value (two-tailed)	0.019	0.019	0.034	0.016
U-87	Correlation coefficient	−0.827 *	−0.817 *	−0.738 *	−0.850 **
	*p* value (two-tailed)	0.011	0.013	0.036	0.008

* Correlation is significant at the 0.05 level (two-tailed). ** Correlation is significant at the 0.01 level (two-tailed).

## Data Availability

All datasets generated for this study are included in the article.
